# One-Bath Pretreatment for Enhanced Color Yield of Ink-Jet Prints Using Reactive Inks

**DOI:** 10.3390/molecules22111959

**Published:** 2017-11-13

**Authors:** Wei Ma, Kezhan Shen, Shuang Li, Meichen Zhan, Shufen Zhang

**Affiliations:** State Key Laboratory of Fine Chemicals, Dalian University of Technology, Dalian 116023, China; Shenkz@mail.dlut.edu.cn (K.S.); shuangli1993@163.com (S.L.); ramblingypsy@126.com (M.Z.)

**Keywords:** ink-jet printing, cotton modification, one-bath pretreatment, reactive dyes

## Abstract

In order to facilely increase the color yield of ink-jet prints using reactive inks, one-bath pretreatment of cotton fabrics with pretreatment formulation containing sodium alginate, glycidyltrimethylammonium chloride (GTA), sodium hydroxide, and urea is designed for realizing sizing and cationization at the same time. The pretreatment conditions, including the concentrations of GTA and alkali, baking temperature, and time are optimized based on the result of thecolor yield on cationic cotton for magenta ink. The mechanism for color yield enhancement on GTA-modified fabrics is discussed and the stability of GTA in the print paste is investigated. Scanning electron microscopey, tear strength, and thermogravimetric analysis of the modified and unmodified cotton are studied and compared. Using the optimal pretreatment conditions, color yield on the cationic cotton for magenta, cyan, yellow, and black reactive inks are increased by 128.7%, 142.5%, 71.0%, and 38.1%, respectively, compared with the corresponding color yield on the uncationized cotton. Much less wastewater is produced using this one-bath pretreatment method. Colorfastness of the reactive dyes on the modified and unmodified cotton is compared and boundary clarity between different colors is evaluated by ink-jet printing of colorful patterns.

## 1. Introduction

Ink-jet printing of textiles has developed rapidly in recent years because of its rapid response to fashion patterns and satisfactory printing effects [1,2,3,4,5,6]. This modern printing is suitable for small-scale production to meet personal demand. In the future, it is largely possible that conventional flat, rotary screen or roll printing techniques will be replaced by ink-jet printing [7,8,9,10]. Cotton fibers, owing to their soft, hygroscopic, and ventilative properties, have a prevailing position in the present developments of textile materials [11,12,13,14,15,16]. Unlike in conventional printing, cotton fabrics need to be pretreated in ink-jet printing with a pretreatment formulation containing a thickener, alkali, and urea, and then dried to remove water prior to ink-jet printing. It is impossible to add regular chemicals into the inks due to special requirements on the ink viscosity, stability, and conductivity [17,18,19,20].

Reactive dye-based inks are commonly used to print cotton and other cellulosic fabrics due to their wide shade range and excellent fastness properties. Due to limited reactivity and hydrolysis of the reactive groups during dye fixation step, reactive dyes only have a fixation of 50–80%, resulting in producing a large amount of dye wastewater [21]. Moreover, monochloro-*s*-triazine dyes with low reactivity are commonly used in ink formulation to meet the requirement for stability, resulting in even low color yield of the prints. Therefore, one of the key problems for ink-jet printing of reactive dyes is the unsatisfactory color yield, which restricts the application of reactive ink-jet printing in producing deep colors [22].

Many attempts have been made to improve the color yield of reactive ink-jet prints. Pretreatment and modification of cotton to increase its reactivity and affinity between the fibers and the dyes were reported to be effective methods, such as pretreatment with gas plasma [1], modification with glycidyltrimethylammonium chloride (GTA) or 3-chloro-2-hydroxypropyltrimethylammonium chloride [14,23,24], dendrimer compounds [4], and chitosan [14,25,26,27,28]. With these methods, the color yield of the prints was improved to a certain extent. Among them, GTA is an effective agent for enhancing color yield. It undergoes an etherification reaction with the primary hydroxyl groups of the fibers, and the hydrolysis side reaction also occurs during the cationization process. GTA is a relatively cheap and low toxic chemical [29]. Moreover, as it is a small molecule, and its influence on dye permeability and colorfastness is low. Kanik et al. [23] used GTA and sodium hydroxide to modify the cotton fabrics at room temperature for 24 h, which were then thoroughly rinsed and dried to remove water from the fabrics. The obtained cationized fabrics were used in ink-jet printing of reactive inks for enhanced color yield. In the study, cationization and pretreatment formulation pretreatment are separated, and the cationization process is long, which makes the whole process complex, and time-and water-consuming. An additional cationization step is disadvantageous in practice, which restricts its real application.

**Figure 1:**
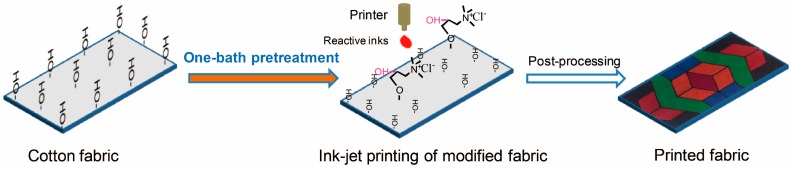
Flowchart of ink-jet printing on modified cotton.

It is found that both GTA cationization and cotton sizing proceed under alkaline conditions. However, the alkali used in the two procedures is different: sodium hydroxide is effective for GTA cationization and sodium carbonate is commonly used for sizing pretreatment. Sodium hydroxide is not conventionally employed in pretreatment formulation because it will promote hydrolysis of the reactive dyes in ink-jet printing and is not beneficial for dye fixation. If the cationization and pretreatment procedures are combined, the process will be simpler and there will be significant time- and water-saving. Thus, in this study, we designed a one-bath pretreatment to combine both GTA cationization and sizing pretreatment. “Sizing” in this paper refers to the surface treatment of cotton with alginate paste, the polymeric component in the pretreatment formulation. With the method, sodium alginate, GTA, sodium hydroxide, and urea are uniformly mixed together in one bath for cotton pretreatment before ink-jet printing. Figure 1 shows the flowchart of the ink-jet printing on the modified cotton. This paper aims to investigate whether this method is effective in improving color yield compared to the previously-reported two-bath method and the influences of the pretreatment parameters, including concentrations of GTA and sodium hydroxide, and baking temperature and time on the color yield. The mechanism for color yield enhancement and the stability of GTA in the pretreatment bath will also be studied. In addition, a comparison will be made on the surface morphology, strength, and thermal properties, colorfastness, and print quality of the cationized and uncationized cotton to comprehensively evaluate the applicability of the one-bath pretreatment method for ink-jet reactive printing.

## 2. Results and Discussion

### 2.1. Effect of Concentration of *GTA* on Color Yield of Magenta Ink

**Figure 2:**
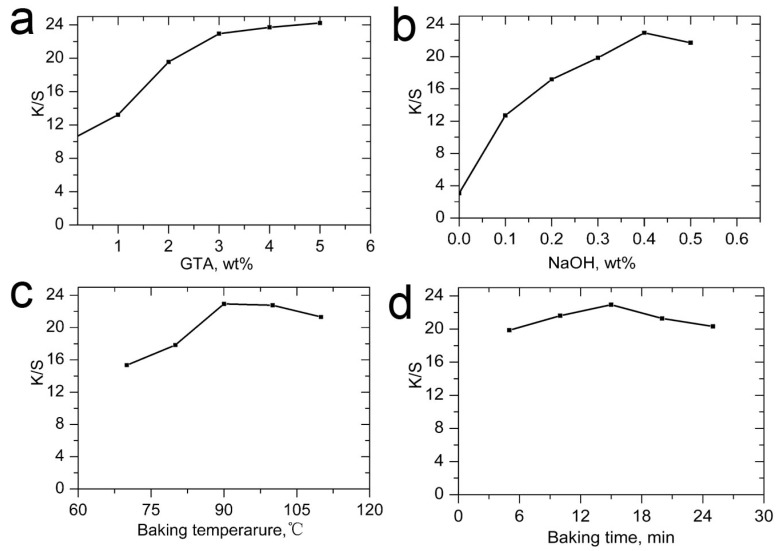
Effects of (**a**) GTA concentration; (**b**) NaOH concentration; (**c**) baking temperature; and (**d**) baking time on the *K/S* values of magenta ink.

In order to investigate the effect of the GTA concentration on *K*/*S*, GTA of different concentrations were used in pretreatment paste and each fabric was ink-jet printed with the magenta ink. The concentration of sodium hydroxide was 0.4 wt % of pretreatment formulation, and all treated cotton was baked at 90 °C for 15 min. The printed samples were steamed for 15 min and then thoroughly washed. The obtained *K*/*S* on the fabrics is shown in Figure 2a.

**Figure 3:**
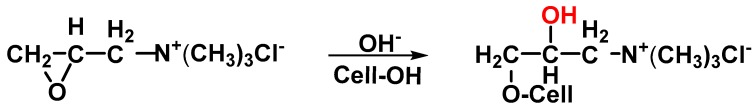
Reaction equation of GTA with cotton fiber.

In Figure 2a, when GTA applied was 0%, 1%, 2%, 3%, 4%, and 5 wt %, *K/S* of the prints was 10.03, 13.23, 19.56, 22.94, 23.71 and 24.22, respectively. *K/S* of the prints increased distinctly with increasing GTA concentration. It can be calculated that *K/S* increased by 31.9%, 95.0%, 128.7%, 136.4% and 141.5% compared with that without addition of GTA when 1%, 2%, 3% and 4% GTA was used, respectively. These results reveal that GTA concentration is a decisive parameter for the increase of the color yield. *K/S* was enhanced significantly especially when the concentration of GTA was higher than 2 wt %. This one-bath pretreatment method can also effectively increase the color yield as the two bath pretreatment method [30]. One reason for *K/S* increase could be higher substantivity of the cationic fibers to the dyes and lower dye penetration in the fibers as demonstrated in previous studies, which was caused by strong ionic attraction between the cationic fibers and the anionic dyes [14,22,27]. The other reason that we considered as the main reason for the *K/S* increase was much higher dye fixation. That means more dyes were covalently bonded to the fibers in the presence of GTA. A color stripping procedure was used to test the covalent bonds between the fibers and the dyes [31]. For the sample with *K/S* of 24.22, *K/S* only showed a small decrease to 23.90 after the color stripping test, indicating most of the dyes were covalently bonded to cotton. All of these results demonstrate that, after pretreatment, the reactivity of the fibers was improved. It is likely that the secondary hydroxyl group, in the 2-position of the propane side chain when GTA reacts with cellulose, is ionized at a much lower pH value than are the cellulose hydroxyl groups and is highly nucleophilic (see Figure 3) [32]. This could be responsible for the higher reactivity. 

In our study, in order to prove the function of the newly introduced hydroxyl group, a contrast experiment was carried out that GTA was first hydrolyzed and then added in the pretreatment formulation. Using the same pretreatment and ink-jet printing processes, we found the *K/S* of the prints did not increase, demonstrating only grafting of GTA onto cotton fibers can enhance fiber reactivity and, finally, realizing a much higher dye fixation. This clearly presents that the main reason for enhancing the color yield were the more reactive hydroxyl groups.

In addition, Figure 2a also shows that when GTA concentration increases from 3% to 5%, the increase of *K/S* was not very high. As *K/S* increased by 128.7% when 3% GTA was used, it is already very beneficial to commercial use, and 3% of GTA was selected to be used in the following investigation.

### 2.2. Effect of NaOH Concentration 

Similar to the conventional printing process, alkali is used in ink-jet printing to ionize the cellulose hydroxyl groups for reactive dye fixation [26]. In ink-jet printing, alkali is added in the pretreatment formulation and pretreated on the cotton fibers together with sizing agent. For cationization of cotton with GTA, alkaline conditions are also required for the reaction. Due to this reason, we to combined sizing and cationization processes in one step. However, it is found that the alkali commonly used in ink-jet printing was not that used for GTA modification. Sodium carbonate is the preferred alkali in reactive dye printing because its alkalinity is suitable for dye fixation [19]. However, for GTA modification, sodium hydroxide is the most effective alkali to promote the reaction. It is always considered that sodium hydroxide accelerates hydrolysis of reactive dyes owing to its strong alkalinity and is not suitable for reactive dyeing and printing.

In this study, we first tried different concentrations of sodium hydroxide for investigation. The effect of NaOH concentration on *K/S* of the prints is shown in Figure 2b. A baking temperature of 90 °C and baking time of 10 min were used for investigation. It can be seen from the figure, when alkali concentration was 0, 0.1%, 0.2%, 0.3%, 0.4%, and 0.5 wt %, *K/S* value was 3.07, 12.70, 17.16, 19.84, 22.94 and 21.71, respectively. *K/S* of the prints increased distinctly with increasing GTA concentration. It can be seen from the data that NaOH addition in pretreatment formulation is very important for the color yield of the ink. Without alkali, the dye almost cannot fix to the cotton, and the color yield is very limited, only 3.07; with a 0.1% alkali addition, the *K/S* significantly increased to 12.70; with a further increase to 0.2%, 0.3% and 0.4%, the *K/S* further greatly increased to 17.16, 19.84 and 22.94, respectively. Addition of NaOH activates the cotton fibers and promotes reaction of GTA with cotton, and further promotes dye fixation. So we can conclude that the *K/S* value is strongly dependent on and very sensitive to the concentration of NaOH. As the concentration further increases from 4% to 5%, *K/S* does not show obvious enhancement, even showing a slight decrease to 21.71. As a further increase in the alkali concentration will accelerate the hydrolysis of reactive dyes and GTA, and show an adverse effect on the formed covalent bonds between the dyes and the fibers, this is not beneficial for achieving a high color yield.

For comparison, 0.4% Na_2_CO_3_ was used instead of NaOH, and the *K/S* value on the modified fabrics reached 17.21, which was much lower than that obtained with the addition of the same concentration of NaOH. From the above investigation, it can be concluded that better fixation of GTA on cotton is a prerequisite for higher dye fixation. As NaOH is more effective for the GTA reaction and dye fixation, it is better than Na_2_CO_3_ in the designed one-bath pretreatment. 

### 2.3. Effect of Baking Temperature

As the reaction temperature shows a significant influence on the reaction of GTA on cotton fibers [14], the baking temperature is studied. According to the references for the reaction of GTA with polysaccharide derivatives, baking temperatures of 70 °C, 80 °C, 90 °C, 100 °C, and 110 °C were chosen for investigation to determine the optimum temperature, the results were shown in Figure 2c. In the study, the concentration of GTA and sodium hydroxide was 3 wt % and 0.4 wt %, respectively. Baking time was 15 min. It could be seen from Figure 2c that *K/S* of magenta ink gradually increased from 15.34 to 22.94 as the baking temperature was raised from 70 °C to 90 °C. However, when the temperature further increased to 100 °C and 110 °C, *K/S* showed slight decrease to 22.78 and 21.32, respectively. As the aim of baking process is to dry the fabrics within a short time, high temperature is usually employed. For the one-bath pretreatment, high temperature baking is also beneficial for reaction between GTA and cotton, so we just chose temperature range of 70 °C to 110 °C. It was found that, for the selected temperature range, the dependence of the color yield on baking temperature is not as high as that on GTA and NaOH concentrations.

### 2.4. Effect of Baking Time on K/S of Magenta Ink

In order to obtain the optimum baking time for the reaction between GTA and cotton fibers, the baking time was set to be 5, 10, 15, 20, and 25 min, and its influence on color yield was shown in Figure 2d. Wherein, the concentration of GTA and sodium hydroxide was 3 wt % and 0.4 wt %, respectively. The baking temperature was 90 °C. From Figure 2d, it can be observed that when the baking time was prolonged from 5 to 15 min, the *K/S* value of the cationized cotton increased from 19.85 to 22.94, respectively. However, when further prolonging the baking time to 20 and 25 min, *K/S* value a little decreased to 21.28 and 20.33, respectively. Further extending the baking time shows adverse effect on the covalent bonds formed, causing a slight decrease in *K/S* of the prints. Based on the results, it could be observed that 5 min is already a good choice for baking time when other conditions have been determined, prolonging the baking time to 15 min can just achieve better results. Accordingly, it can be concluded that the color yield of the dye is much less sensitive to baking time compared with other conditions.

Based on above data analysis, it could be summarized that *K/S* value of the dye is strongly dependent on the concentrations of GTA and NaOH, moderately dependent on baking temperature and much less dependent on baking time. According to the data in Figure 2, we chose 3 wt % GTA, 0.4 wt % NaOH, baking temperature of 90 °C, and baking time of 15 min as the optimized conditions for one-bath pretreatment application.

### 2.5. Stability of the Pretreatment Agent

**Figure 4:**
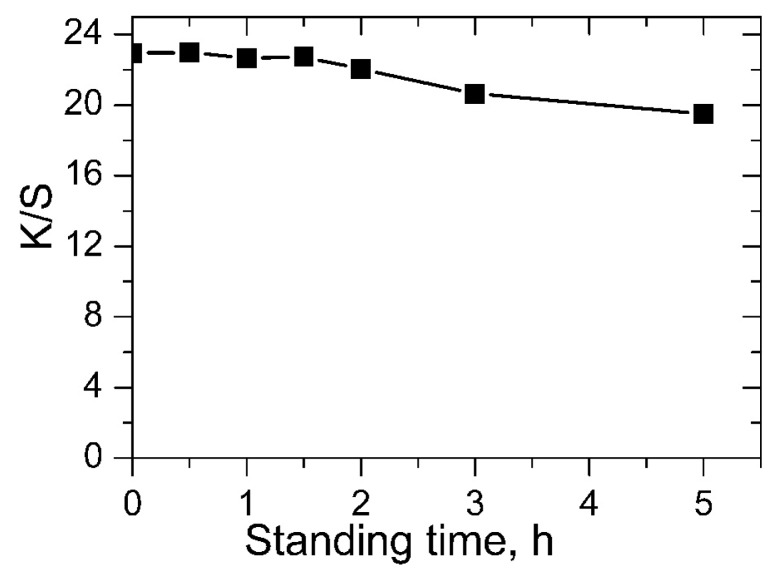
*K/S* values of the prints when pretreatment bath standing for 0, 0.5, 1, 1.5, 2, 3, and 5 h.

In industrial production, the stability of the pretreatment agent is essential for the stability of the ink-jet printing. The agent with excellent stability contributes to the continuity of production and prolongs the pretreatment agent replacement cycle. GTA is sensitive to alkali due to existence of its epoxy group. In the designed one-bath pretreatment method, GTA and NaOH are dissolved together in the pretreatment formulation, so the stability of GTA in the pretreatment formulation is an important factor to be evaluated. The baths were placed for different time before usage. *K/S* values of the prints with different pretreatment baths were measured and the results were presented in Figure 4. It shows that the color yield of the prints almost does not change when standing time was 0, 0.5, 1 and 1.5 h at room temperature, the *K/S* values of prints was 22.94, 23.00, 22.65, 22.73, respectively. Then *K/S* values experienced a slight decrease to 22.04 after standing for 2 h. A further increase of standing time to 3 h further showed decrease in *K/S* value to 20.64. Thus, with this one-bath pretreatment method, it is better to use the bath within 2 h to maintain the continuity of production, and NaOH should be added just before the pretreatment process.

### 2.6. Surface Morphology 

**Figure 5:**
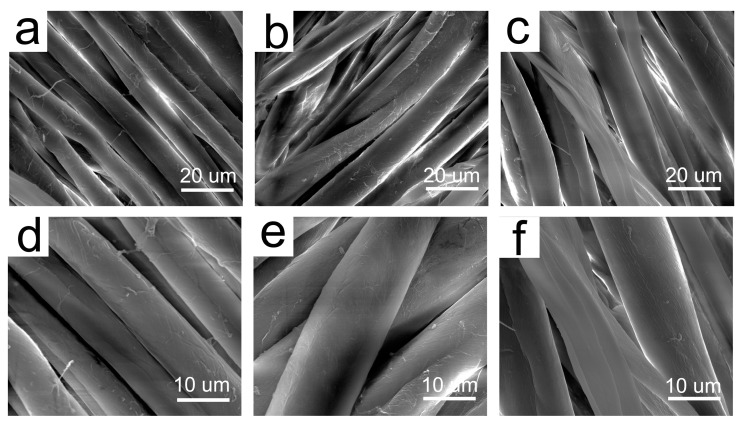
SEM images of different cotton fibers: (**a**) raw cotton, ×3000; (**b**) uncationized cotton, ×3000; (**c**) cationized, ×3000; (**d**) raw cotton, ×6000; (**e**) unmodified, ×6000; and (**f**) modified, ×6000.

In order to study the influence of cationization on the surface morphology of the fibers, the cotton was dip-padded pretreatment formulation, with and without GTA, and baked at 90 °C for 15 min, and then rinsed thoroughly. The surface morphology of thus-obtained uncationized and cationized cotton fibers was examined by SEM (see Figure 5). Additionally, the SEM of the raw fibers was also shown for comparison. It can be seen no obvious difference among the raw, uncationized and cationized cotton fibers. There seems to be some impurity on the raw fibers, the surfaces of the uncationzied and cationized cotton fibers seem much cleaned after pretreatment under alkaline and high-temperature conditions, and rinsing. No damage was detected on the surface of alkali-treated and GTA-treated fibers, indicating the pretreatment conditions did not show any adverse effect on the surface morphology of the cotton fibers.

### 2.7. Tear Strength

Tear strength of cotton fabrics was measured to investigate the mechanical strength of the fabrics (Table 1). Table 1 shows the tear strength of the raw fabric is 12.4 N in warp and 10.2 N in weft, that of the uncationized cotton is 12.2 N in warp and 9.8 N in weft, indicating a slight decrease in tear strength after alkali and high-temperature treatment. For the cationized fabric, its tear strength presents further decrease to 12.1 N in warp and 9.5 N in weft. Even though the tear decrease of the cationic cotton is quite small compared to that of the uncationized one, it can still show a certain influence of the pretreatment with GTA.

### 2.8. Determination of Thermal Properties

**Figure 6:**
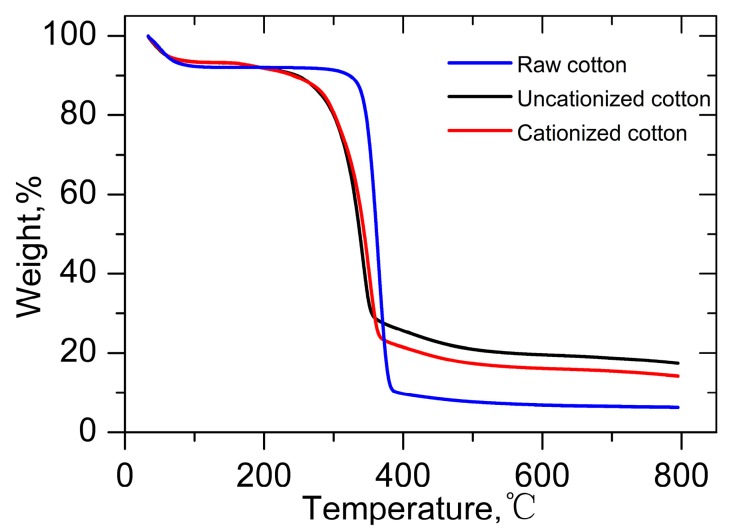
Thermogravimetric analysis of raw, uncationized, and cationized cotton.

Thermogravimetric analysis was used to investigate the thermal properties of different cotton fabrics. Figure 6 presents the thermal degradation of the raw, uncationized, and cationized cotton. The stability of the raw cotton was better than the cotton treated with pretreatment formulation as a significant weight loss happened at 340 °C for raw cotton, while at 290 °C for uncationized and cationized cotton. This difference may be due to the corrosion effect of NaOH on cotton under high baking temperature. However, no distinct change was detected between uncationized and cationized fabrics. This result indicated that the effect of GTA on the thermal property of cotton was not obvious. All cotton samples began to produce char at higher temperature. Carbon and charred residues increased in this process because of dewatering and charring reactions [33,34].

### 2.9. Effect of GTA Modification on Different Colors of Ink and Colorfastness

**Figure 7:**
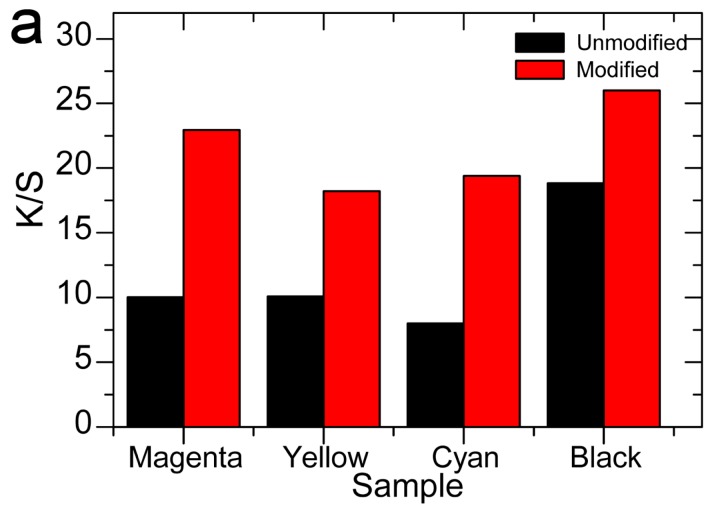

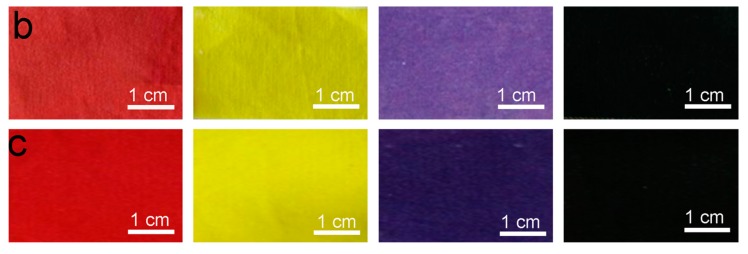
(**a**) *K/S* values on uncationized and cationized cotton with magenta, yellow, cyan, and black inks; and digital photos of uncationized (**b**) and cationized (**c**) cotton printed with magenta, yellow, cyan, and black inks.

**Figure 8:**
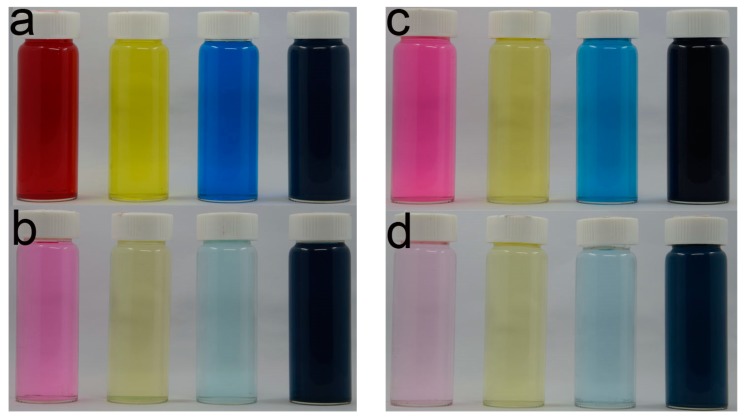
Washing wastewater of the ink-jet printed uncationized (**a**); and cationized cotton (**b**). Soaping residue of the ink-jet printed uncationized (**c**); and cationized cotton (**d**) (uncationizedand cationized cotton were washed and soaped under the same conditions. The volume of water was 200 mL for washing and 120 mL for soaping).

Magenta, yellow, cyan, and black inks are the four basic colors for ink-jet reactive printing in this study. They are ink-jet printed on cotton pretreated with and without GTA under the above optimal conditions to show the applicability of the one-bath pretreatment method. The digital pictures of the prints and the washing and soaping wastewater are presented in Figure 7 and Figure 8, respectively. From Figure 7, the distinct *K/S* increase of the cationized fabrics for all four colors can be observed. The contrast of washing and soaping wastewater between the uncationized and cationized fabrics is quite obvious in Figure 8, revealing much less dye wastewater is produced with the designed one-bath pretreatment method. Although the comparison of soaping residue (See Figure 8c,d) is not as significant as that of washing waste water(See Figure 8a,b), the difference between the uncationized (Figure 8c) and cationized (Figure 8d) results is still easily detected for all the colors. The results demonstrate that higher dye fixation is achieved and much less dye wastewater is produced with the one-bath pretreatment method, which greatly reduces the adverse effect of ink-jet reactive printing on environment. Accordingly, it is considered that the one-bath pretreatment is a much cleaner and water-saving process.

Color yield, and colorimetric and colorfastness properties of the prints are listed in Table 2. *K/S* values of all four prints on modified cotton were much higher. L values of the prints on the cationic cotton were lower than the corresponding values on the uncationized one, indicating that the color looks darker. There were also changes in redness-greenness (a) and yellowness-blueness (b) for different dyes. One of the main reasons for the colormetric difference is the different color yield on the fabrics; however, it is difficult to account for the direct effect of the cationic quanternary ammonium groups on the color of the dyes associating with them. This may suggest some difference in the physical environment of the dyes on the modified and unmodified fabrics. For practical purposes, the color difference could be accommodated in the formulation of the ink recipes.

Table 2 also shows that wash, crock and light fastness properties of the inks on cationized and uncationized fabrics. The wash fastness of the inks on the cationic cotton was all assessed as good, reaching 4 or 4–5 grade. Dry and wet crock fastness of the inks on the cationized fabrics was also comparable with that obtained from uncationized ones. In the case of magenta and yellow inks, the wet fastness on the cationic cotton was 1 and 0.5 grade higher, respectively. Moreover, light fastness of the prints on the cationic cotton was much satisfactory. For cyan, magenta, and yellow inks, the light fastness was even 0.5, 1 and 1.5 grades higher with the one-bath pretreatment method. The above results demonstrate that the designed pretreatment method has a good effect on the colorfastness of the inks.

### 2.10. Colorful Pictures 

**Figure 9:**
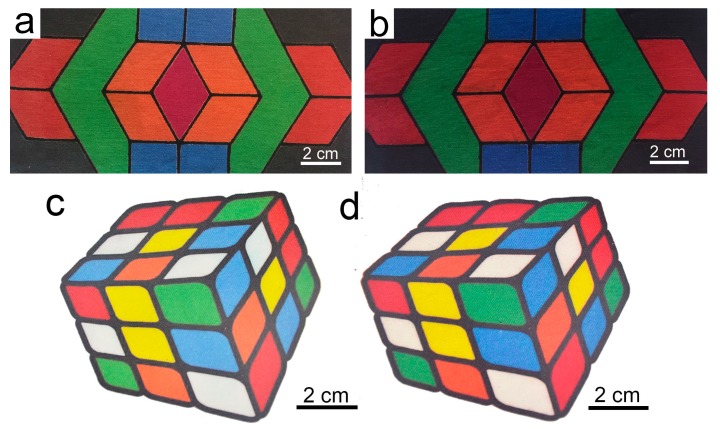
Colorful pictures of ink-jet printing on uncationized (**a**,**c**) and cationized cotton (**b**,**d**).

A flat colorful picture and a three dimensional magic cube (Figure 9) were ink-jet printed on both uncationized and cationized fabrics to study the color and boundary properties. For each color of the pictures, it is produced from several colors, a result of color match. It can be clearly seen from Figure 9a,b that the red, orange, green, blue, and black colors are all much deeper on the modified cotton. The boundary between different colors is very clear. For the picture of the three dimensional magic cube in Figure 9c,d, the small squares with red, orange, green, and blue colors all look deeper on the modified cotton, while the squares with yellow color do not look that deeper due to that it is a much lighter color, and all white square parts are neat. The whole picture on the cationic cotton bring us visual enjoyment. From the above results, it can be concluded that the one-bath pretreated cotton fabrics shows very good applicability in ink-jet printing of colorful pictures. Color difference produced with this method can be corrected by making some adjustment in the ink formulation or ink-jet printing equipment.

## 3. Experimental

### 3.1. Materials

Bleached, desized, and mercerized 100% cotton (150 g/m^2^), was purchased from Test Fabrics, Inc. (Shanghai, China). Magenta, yellow, cyan, and black reactive dye inks were supplied by Fancell International Co. (Shanghai, China). Glycidyltrimethylammonium chloride (GTA) was purchased from Saen Chemical Technology Co. (Shanghai, China). Sodium hydroxide (NaOH) was purchased from Tianli Chemical Reagent Co. (Tianjin, China). Urea was obtained from Damao Chemical Reagent Co. (Tianjin, China). Sodium carbonate anhydrous was purchased from Bodi Chemical Reagent (Tianjin, China). All the solvents were analytically pure. Sodium alginate was obtained from Runlong Marine Biopharmacy Co. (Yantai, China), which is a medium-viscosity, printinganddyeing-class.

### 3.2. Pretreatment of Cotton Fabrics

One-bath pretreatment of cotton fabrics was carried out with a dip-pad procedure. The printing phase was prepared by first mixing a certain amount of sodium hydroxide, 1.25 g of sodium alginate and 2.5 g of urea, and added deionized water to weight of 40 g in total. The mixture was then heated to 50 °C under stirring for dissolving. After that, 10 g GTA solution, which was obtained by dissolving a certain amount of GTA in deionized water, was mixed uniformly with the pretreatment formulation before use. In the control pretreatment bath, no GTA was added. The cotton fabrics were dipped in the pretreatment bath, and then padded using a two-roll padder (MU 505T; Beijing Textile Equipment Institute, Beijing, China) with a 0.3 MPa roll pressure to give a wet pick-up of 80%. The fabrics were subsequently baked at a certain temperature for some time using a rapid baker. Baking is an important step in pretreatment for drying the cotton and promoting the reaction between cotton fibers and GTA. Then the treated fabrics were ready for ink-jet printing. 

In our experiment, alternative cationic agent-hydrolyzed GTA was also used in the one-bath pretreatment of cotton fibers for comparison for mechanism study. GTA was hydrolyzed in 12.5% of Na_2_CO_3_ solution at 50 °C for 40 min. The pH of the solution was then adjusted to neutral and ready to use. The procedure for preparation of the pretreatment formulation containing hydrolyzed GTA was the same as that of GTA-containing paste; only hydrolyzed GTA solution was used, instead of the GTA solution. 

### 3.3. Ink-Jet Printing

A square 3 × 5 inch picture was printed with an ink-jet printer (L310; Epson, Suwa, Japan) at a resolution of 5760 × 1440 dpi for each single color. Color parameters (RGB) were (255, 0, 0) for magenta, (255, 255, 0) for yellow, (0, 0, 255) for cyan, and (0, 0, 0) for black. After being printed, the samples were air-baked and then steamed for 15 min.

### 3.4. Wash-Off Procedure

The printed and steamed cotton fabrics were consecutively washed with cold water, warm water, and cold water. They were then soaped in a 0.1 wt % soap solution at 95 °C with a goods-to-liquor ratio of 1:30 for 10 min, and eventually washed thoroughly with cold water and warm water, and then air dried. For color stripping, a four-step process was employed using Höechst agents of (1) acetic acid and ethanol (1:1, *v*/*v*); (2) 1 wt % aqueous ammonia; (3) a mixture of *N*,*N*-Dimethylformamide (DMF) and water (1:1, *v*/*v*); and (4) DMF. In each step, the samples were boiled in the each agent for 4 min, and rinsed with cold water between the steps. 

### 3.5. Measurement of Color Yield

The color yield (*K/S*) of the ink-jet printed fabrics were measured using a UltraScan XE color Measuring and Matching Meter (HunterLab Co., Reston, VA, USA) at room temperature. The color yield ranging from the wavelength of 400 nm to 700 nm with 10 nm interval was measured and calculated using Equation (1) [14]. All *K/S* values were determined at λ_max_ and average values were obtained at five different positions for each cotton:

(1) KS=(1−R)22R

where *K* is the light absorption coefficient of the fabric, *S* is the light scattering coefficient, and *R* is the light reflectance at the maximum wavelength of the fabric.

### 3.6. Determination of Nitrogen Content

The samples were dried under vacuum at room temperature for 24 h before measurement. Nitrogen content of cationic cotton was determined by the Kjeldahl method with a KDY-9820 Kjeldahl nitrogen apparatus (Tongrun Electrical and Mechanical Technology Co., Ltd., Beijing, China). For each condition, nitrogen content of three samples were measured to obtain the average. In this study, the nitrogen content of the uncationized and cationized cotton obtained under the optimal conditions was 0.58 wt % and 0.77 wt %, respectively.

### 3.7. Scanning Electronic Microscopy

The surface morphology of the pretreated fibers was investigated using a Quanta 200 (FEI instruments company, Hillsboro, OR, USA) scanning electron microscope (SEM). The cotton fiber samples were coated with gold sputtering at room temperature before measurement.

### 3.8. Thermogravimetric Analysis

The thermal degradation property of the samples was performed on a Q500 thermoanalyzer from the TA instrument company (New Castle, DE, USA). A certain weight of sample was tested under N_2_ at a heating rate of 10 °C/min with the temperature ranging from 30 °C to 800 °C.

### 3.9. Tear Strength Testing

The tear strength of the printed cotton fabrics was tested according to ASTM D 5734-1995 using a YG(B)033A tearing instrument (Wenzhou, China). Each sample was tested three times and the average value was used.

### 3.10. Colorfastness Properties

The wash fastness of the reactive prints on cotton was tested according to AATCC Test Method 61-2001 with an S-1002 two-bath dyeing and testing apparatus (Roaches Co., West Yorkshire, UK). The crock fastness was tested according to AATCC Test Method 8-2001 using Y(B)571-II crockmeter (Darong Standard Textile Apparatus Co. Ltd., Wenzhou, China). The light fastness was tested according to AATCC Test Method 16-2001using a YG(B)611-V lightfastness tester (Darong Standard Textile Apparatus Co. Ltd., Wenzhou, China) [35].

## 4. Conclusions

One-bath pretreatment of cotton fabrics with a GTA-containing pretreatment formulation is effective in improving the color yield of the reactive ink-jet prints. Sodium hydroxide, instead of sodium carbonate, in the pretreatment formulation can promote both GTA and reactive ink fixation. Optimal pretreatment conditions for magenta ink was 3 wt % GTA, 0.4 wt % NaOH, baking temperature of 90 °C, and baking time of 15 min. With these conditions, *K/S* on the cationic cotton for magenta, cyan, yellow, and black reactive inks are 22.94, 18.22, 19.40 and 26.01, respectively. These results are increased by 128.7%, 142.5%, 80.9% and 38.1%, respectively, compared with the corresponding color yield on the uncationized cotton. All colorfastness of the dyes are satisfactory, and lightfastness of magenta, yellow, and cyan inks on the cationic cotton is even higher than that on the uncationized one. Colorful ink-jet prints with this one-bath pretreatment method exhibit higher color yield and good boundary clarity. Therefore, using one-bath pretreatment for reactive ink-jet printing has considerable potential in terms of high dye utilization ratio and reduction in effluent. Further work is underway to investigate how to increase the stability of GTA in the pretreatment formulation for reactive ink-jet printing. 

## Figures and Tables

**Table 1:** Tear strength of raw, uncationized, and cationized cotton.

Cotton	Warp (N)	Weft (N)
Raw	12.4	10.2
Uncationized	12.2	9.8
Cationized	12.1	9.5

**Table 2:** Colorfastness and the L, a, b of the four kinds of inks printed on cotton fabrics treated in the absence and presence of GTA, respectively.

Color	Cotton	*K/S*	L	a	b	Wash Fastness			Crock Fastness		Light Fastness
						Change	Staining		Dry	Wet	
							Cotton	Wool			
Magenta	I	10.03	39.86	47.95	23.90	4–5	4–5	4–5	4–5	3–4	3–4
	II	22.94	35.91	48.60	19.57	4–5	4–5	4–5	4–5	4–5	5
Yellow	I	10.07	84.00	−11.28	87.16	5	4–5	4–5	4–5	4	5
	II	18.22	79.07	−13.27	82.53	4–5	4	4–5	4–5	4–5	6
Cyan	I	8.00	34.11	13.37	−31.46	4–5	4–5	4–5	4–5	4	3
	II	19.40	24.72	12.37	−32.09	4–5	4–5	4–5	4–5	3–4	3–4
Black	I	18.83	18.30	–0.39	−3.54	4–5	4–5	4–5	5	4	4
	II	26.01	15.96	0.11	−3.45	4–5	4–5	4–5	4–5	3–4	4

I: Cotton treated in the absence of GTA; II: Cotton treated in the presence of GTA.
